# First report of a novel *Paenibacillus* strain JDF1 and its biocontrol potential against citrus bacterial canker

**DOI:** 10.3389/fmicb.2026.1740692

**Published:** 2026-02-12

**Authors:** Ze-Qiong Wang, Yu-Xiong Xiao, Zhu Tong, Xiu-Juan He, Hai-Yue Zhang, Wen-Ming Qiu

**Affiliations:** Hubei Key Laboratory of Germplasm Innovation and Utilization of Fruit Trees, Institute of Fruit and Tea, Hubei Academy of Agricultural Sciences, Wuhan, China

**Keywords:** antibiotic substance, biocontrol, citrus bacterial canker, genome analysis, *Paenibacillus*

## Abstract

Citrus bacterial canker (CBC), caused by *Xanthomonas citri* subsp. *citri* (*Xcc*), causes severe annual yield losses on citrus crops worldwide. Current control strategies including quarantine, copper-based chemicals, and resistant varieties are limited by poor adaptability, pathogen resistance, and long breeding cycles. Herein, a novel endophytic *Paenibacillus* strain, designated JDF1, was isolated from *Veronica persica* (a common weed in citrus orchards). *In vitro* assays demonstrated that JDF1 inhibited *Xcc*. Greenhouse trials with “Eureka” lemon showed that low-concentration JDF1 (OD_600_ = 0.2) and its cell-free supernatant eliminated CBC symptoms without inducing phytotoxicity. Notably, JDF1 retained over 60% of its activity following heat treatment at 50 °C for 6 h. Phylogenetic analyses revealed that JDF1 is closely related to *P. xylanexedens* (98.52% 16S rRNA gene similarity) and *P. polysaccharolyticus* (99.8% housekeeping gene similarity). Further average nucleotide identity (ANI) analysis confirmed that strain JDF1 belongs to the same species as *P. polysaccharolyticus*, with a sequence similarity of 97.31%. JDF1 tolerated CaCuSO_4_ concentrations up to 600 mg/L and exhibited resistance to ampicillin and streptomycin. Genome sequencing of JDF1 predicted multiple genes associated with antibiotic biosynthesis, primarily including those involved in the production of polyketide, terpenoid, tyrocidin A, iturin A, lanthipeptide (class II), ranthipeptide, and lassopeptide. In a deeper investigation of the biocontrol mechanisms, a phospholipid antibiotic, bacilysocin, was identified in the JDF1 culture via LC-MS analysis. Collectively, these findings indicate that JDF1 is a promising novel biocontrol agent (BCA) against *Xcc*, with the dual advantages of high efficacy at low concentrations and thermostability, thereby enhancing its potential for agricultural application.

## Highlights

- First report of a novel *Paenibacillus* strain JDF1 exhibiting antagonistic activity against the citrus canker pathogen.
- Low concentrations (OD_600_ = 0.2) of JDF1 and its supernatant can effectively control the occurrence of citrus canker.
- High concentrations (OD_600_ = 1.0) of JDF1 induce a necrotic response in citrus leaves and retain approximately 60% antibacterial activity after treatment at 50 °C for 6 h.
- JDF1 exhibits tolerance to CaCuSO_4_ and resistance to antibiotics such as streptomycin.
- Genomic and metabolomic analyses suggest that JDF1 may produce multiple antimicrobial substances, such as tyrocidin A, iturin A and bacilysocin.

## 1 Introduction

Citrus is one of the most economically important fruit crops globally, cultivated across tropical and subtropical regions, with significant contributions to agricultural livelihoods, food security, and human nutrition (e.g., vitamin C supply). However, the sustainable development of the citrus industry is severely threatened by citrus bacterial canker (CBC), a highly destructive disease caused by the Gram-negative bacterium *Xanthomonas citri* subsp. *citri* (*Xcc*). *Xcc* invades host plants through natural openings (e.g., stomata) or wounds, colonizes the apoplast, and induces raised, corky lesions on leaves, twigs, and fruits. Meanwhile, *Xcc* exhibits strong environmental persistence (surviving in plant debris, soil, or asymptomatic hosts), which complicates eradication efforts and facilitates long-distance spread via contaminated materials (Shahbaz et al., 2023).

Existing strategies for CBC control rely primarily on three approaches, each with critical limitations. First, physical approaches (e.g., inspection/destruction of infected plants, periodic inspection of citrus orchards, and establishment of windbreaks) prevent initial *Xcc* introduction but fail in pathogen-established regions; In addition, planting resistant varieties is an important method for controlling citrus canker, while the commercial constraints of these varieties restrict the practicality of this strategy (Shahbaz et al., 2023; Graham et al., 2004). Second, chemical control—dominated by copper-based formulations and antibiotics (e.g., streptomycin)—is widely used but problematic: *Xcc* has evolved copper resistance in Brazil, China, and the U.S. (Richard et al., 2017; McManus et al., 2002); antibiotic overuse raises cross-resistance risks in human pathogens and regulations (e.g., EU's EC No 396/2005; https://eur-lex.europa.eu/legal-content/EN/TXT/?uri=CELEX:32005R0396) restrict its application. Additionally, chemicals harm non-target microorganisms and cause environmental accumulation (Behlau et al., 2012; Carvalho et al., 2020). Third, biological control includes genetic and transgenic breeding, as well as microbial inoculants; the former is hindered by limited natural resistance genes in commercial germplasm and long breeding cycles (10–15 years). Transgene-free genome editing techniques, such as CRISPR/Cas9 targeting the *lob1* locus, hold considerable promise for crop improvement, as demonstrated by early proof-of-concept studies (Jia et al., 2016). However, their path to commercialization is hampered by significant regulatory hurdles and public acceptance concerns, which are well-documented barriers for new agricultural technologies. The latter has emerged as an environmentally friendly alternative, which utilizes beneficial microorganisms or their metabolites to suppress *Xcc* via antibiosis, niche competition, induced systemic resistance (ISR), or virulence factor degradation (Villamizar et al., 2020; Luo et al., 2026). Recent advances include screening biocontrol agents (BCAs) such as *Pseudomonas* spp. (Michavila et al., 2017), actinomycetes (Rodrigues et al., 2018), fungi (Vieira et al., 2018), bacteriophages (Ahmad et al., 2014), and *Bacillus* spp. (Qian et al., 2021). Among these, *Bacillus* spp. secrete diverse antimicrobial secondary metabolites: cyclic lipopeptides (surfactin, fengycin), polyketides (difficidin, bacillaene), and dipeptides (bacilysin), which target *Xcc* via membrane disruption or metabolic inhibition (Qian et al., 2021; Wang et al., 2022; Rabbee and Baek, 2023). Beyond direct antibiosis, *B. velezensis* strains further inhibit *Xcc* by downregulating virulence genes (e.g., type III secretion system genes) via cell-free supernatants (Kenfaoui et al., 2024). *Bacillus* spp. enhance citrus resistance via ISR—upregulating defense genes (e.g., *pr*; Luo et al., 2026). However, despite its phylogenetic relatedness to *Bacillus* spp., the ability of *Paenibacillus* spp. to control citrus canker has not been documented to date.

*Paenibacillus* spp., closely related to *Bacillus*, share spore-forming capabilities and exhibit comparable biocontrol potential. Widely distributed in agricultural soils and plant tissues, *Paenibacillus* spp. produce antimicrobials such as polymyxins, fusaricidins, and paenicidins (Vater et al., 2015; Lebedeva et al., 2021). Strain UY79 of *Paenibacillus* spp., isolated from an *Arachis villosa* root nodule, exhibits broad-spectrum antifungal activity (Costa et al., 2022). Polymyxin B1 and E2 produced by *P. polymyxa* Y-1 exhibit efficacy against rice bacterial diseases (Yi et al., 2022). Research on the application of *Paenibacillus* spp. in citrus disease control is relatively limited. *Paenibacillus* strain YS-1 has been reported as a promising biocontrol agent against penicillium decay of “Nanfeng” mandarins (Tu et al., 2013). However, its potential for managing citrus canker has not been reported.

In this study, we isolated a novel *Paenibacillus* strain (designated JDF1) from leaves of *Veronica persica*. JDF1 is phylogenetically related to *P. polysaccharolyticus*. We verified JDF1's antagonistic activity against *Xcc*, evaluated its thermostability, and assessed its CBC control efficacy in greenhouse-grown citrus seedlings. Co-culture assays confirmed JDF1's tolerance to calcium copper sulfate (≤600 mg/L) and resistance to ampicillin/streptomycin, highlighting its potential for integrated CBC management. Genome sequencing of JDF1 identified biosynthetic gene clusters (BGCs) linked to antimicrobial metabolites, including polyketide, terpenoid, tyrocidin A, iturin A, lanthipeptide (class II), ranthipeptide, and lassopeptide. LC-MS-based metabolomics analysis further revealed the presence of bacilysocin, a phospholipid antibiotic, in strain JDF1. Integrating genomic and metabolomic data, we preliminarily elucidated the potential antimicrobial metabolites and mechanisms of JDF1. Our results demonstrate that JDF1 exhibits strong, thermostable antagonism against *Xcc*, suppresses CBC with low phytotoxicity, and produces unique antimicrobial metabolites—positioning it as a promising BCA for sustainable CBC management.

## 2 Materials and methods

### 2.1 Isolation and screening of strains

The leaves of *Veronica persica* (*Veronica persica* Poir) were collected from a citrus orchard at the Fruit and Tea Research Institute, Hubei Academy of Agricultural Sciences, Wuhan City, Hubei Province, China (30.29° N, 114.18° E) and thoroughly rinsed with sterile distilled water to remove surface contaminants. In a laminar flow hood, the cleaned leaves were immersed in 75% (v/v) ethanol for 30 s for surface sterilization, followed by three successive rinses with sterile distilled water to eliminate residual ethanol. Subsequently, 3–5 leaf segments (approximately 1 cm^2^ each) were excised and transferred into a 1.5 mL microcentrifuge tube containing 1 mL of sterile distilled water. The tube was vortexed vigorously to homogenize the leaf tissues, and the homogenate was allowed to stand at room temperature for 30 min to facilitate the release of endophytic bacteria. A 100 μL aliquot of the supernatant was pipetted and evenly spread onto Luria-Bertani (LB) agar plates (tryptone 10 g/L, yeast extract 5 g/L, NaCl 10 g/L, agar 15 g/L, pH 7.0). Each treatment was performed in triplicate, and the plates were incubated at 28 °C for 24 h. After incubation, colonies with distinct morphological characteristics (e.g., color, shape, margin, and texture) were selected and individually inoculated into LB liquid medium for activation. The activated bacterial strains were preserved at −80 °C in LB medium supplemented with 20% (v / v) glycerol for long-term storage.

### 2.2 Screening and identification of antagonistic strains

#### 2.2.1 Plate antagonism assay for screening antagonistic strains

*Xcc* strain (DL-509 labeled with enhanced green fluorescent protein; GenBank accession number: CP030178.1) was provided by the Laboratory of Changsha Branch, National Citrus Improvement Center. It was cultured in LB liquid medium at 28 °C with shaking (180 rpm) until the bacterial concentration reached 1.0 × 10^8^ CFU/mL. A 100 μl aliquot of the *Xcc* suspension was uniformly spread onto LB agar plates and allowed to air-dry in a laminar flow hood. A grid pattern (3 × 3) was marked on the bottom of each plate to guide bacterial spotting. For each isolated bacterium, a 2 μl aliquot of the activated bacterial suspension (1.0 × 10^8^ CFU/ml) was spotted onto each grid intersection. The plates were incubated at 28 °C for 48 h and the presence and diameter of inhibition zones around each spotted strain were measured to evaluate antagonistic activity against *Xcc*. A total of approximately 1,000 isolates were screened, and 9 strains showing clear inhibition zones were selected for further analysis.

#### 2.2.2 Pathogenicity test on citrus leaves

To exclude strains with potential pathogenicity to citrus, the 9 antagonistic strains were subjected to a citrus leaf pathogenicity assay. Lemon (*Citrus limon* (L.) Burm. f. cv. Eureka) seedlings (6-month-old) were used as test plants. Five seedlings were assigned to each treatment group, with one to two young leaves sampled from each seedling. From these pooled leaves, five were randomly selected for subsequent observation and testing. Bacterial suspensions of each strain were adjusted to 1.0 × 10^8^ CFU/mL with sterile distilled water. Lemon leaves were gently pricked with a sterile needle to create small wounds, and 10 μl of the bacterial suspension was inoculated onto the wound sites. Sterile distilled water was used as a negative control, and *Xcc* suspension (1.0 × 10^8^ CFU/ml) was used as a positive control. The inoculated seedlings were placed in a growth chamber under controlled conditions (24 °C, 75% relative humidity, 16 h light/8 h dark cycle, approximately 180 μmol photon m^−2^ s^−1^ light intensity) and incubated for 15 days. The incidence of disease symptoms (e.g., yellow halos, callus-like eruptions, or necrosis) on the leaves was recorded daily. Among the 9 strains, 7 strains showed strong pathogenicity to lemon leaves (manifested as severe necrosis and lesion expansion), while the other 2 strains exhibited no pathogenicity. The tests were repeated at least three times, and one of the non-pathogenic strains, designated JDF1, showed rapid growth on LB plates (forming visible colonies within 12 h) and produced a viscous biomass that was easy to scrape. This strain (JDF1) was selected for subsequent experiments.

#### 2.2.3 Morphological identification of strain JDF1

Strain JDF1 was inoculated onto LB agar plates and incubated at 28 °C for 48 h. The morphological characteristics of the colonies, including color, shape, margin, transparency, and texture, were observed and photographed using a stereomicroscope (Olympus SZX16, Japan). For cell morphology analysis, JDF1 cells were stained using the Gram staining method. Briefly, bacterial smears were prepared on glass slides, heat-fixed, and stained with crystal violet (1 min), iodine solution (1 min), 95% ethanol (decolorization for 30 s), and safranin (1 min). The stained smears were observed under a light microscope (Olympus BX53, Japan) at 1,000× magnification to determine cell shape, arrangement, and Gram staining reaction.

#### 2.2.4 16S rDNA sequencing and phylogenetic analysis

Genomic DNA of strain JDF1 was extracted using the TIANamp Bacteria DNA Kit (Tiangen Biotech, Beijing, China) according to the manufacturer's instructions. The 16S rDNA gene was amplified using the universal bacterial primers 27F (5′-AGAGTTTGATCCTGGCTCAG-3′) and 1492R (5′-TACGGCTACCTTGTTACGACTT-3′). The PCR was performed in a 50 μl volume containing 25 μl of 2 × Taq PCR MasterMix (Tiangen Biotech, Beijing, China), 2 μl of each primer (10 μmol/L), 2 μl of genomic DNA template (50 ng/μl), and 19 μl of sterile distilled water. The PCR cycling conditions were as follows: initial denaturation at 98 °C for 3 min; 45 cycles of denaturation at 98 °C for 10 s, annealing at 55 °C for 15 s, and extension at 72 °C for 20 s; and a final extension at 72 °C for 5 min. The PCR products were separated by 1.0% (w / v) agarose gel electrophoresis (120 V, 25 min) and visualized under a UV transilluminator. The target band (approximately 1,500 bp) was excised and purified using the TIANgel Midi Purification Kit (Tiangen Biotech), then sent to Tianyi Huayu Gene Technology Co., Ltd. (Wuhan, China) for Sanger sequencing.

The obtained 16S rDNA sequence (1438 bp) was compared with sequences in the GenBank database using the BLAST program (https://blast.ncbi.nlm.nih.gov/Blast.cgi). Homologous sequences of closely related strains were downloaded, and a phylogenetic tree was constructed using MEGA 11 software with the neighbor-joining (NJ) method (1,000 bootstrap replicates). The Average Nucleotide Identity (ANI) between the genome of strain JDF1 and closely related type strains was calculated to delineate taxonomic boundaries. Genome sequences of reference strains were retrieved from the NCBI RefSeq database. ANI values were determined using FastANI v1.34 with default parameters (kmer size = 16, fragment length = 3,000 bp). The analysis was performed in a bidirectional manner, and the resulting ANI values were reported as the mean of both directions. A threshold of ≥95% ANI was applied as the criterion for species assignment. The analysis was conducted on a high-performance computing cluster, and the output was visualized using R v4.3.1.

### 2.3 Evaluation of biocontrol efficacy of JDF1 against citrus bacterial canker

#### 2.3.1 Biocontrol efficacy of JDF1 bacterial suspension

“Eureka” lemon seedlings (6-month-old) were used for the *in vivo* biocontrol assay. Five independent seedling lines of “Eureka” lemon were selected for each treatment, and one to two young leaves were subjected to experimental treatment from each line. Subsequently, one treated leaf per line was randomly chosen as one replicate, and each treatment thus comprised five biological replicates. Leaf disk (0.5 cm in diameter) were collected from the inoculation sites using a sterile punch and used for the determination of *Xcc* bacterial titers. Strain JDF1 was cultured in LB liquid medium at 28 °C with shaking (180 rpm) until the optical density at 600 nm (OD_600_) reached 0.2 or 1.0. *Xcc* was cultured under the same conditions until the concentration reached 1.0 × 10^8^ CFU/mL. For the treatment group, equal volumes of JDF1 suspension (OD_600_ = 0.2 or 1.0) and *Xcc* suspension were mixed; for the control group, equal volumes of sterile distilled water and *Xcc* suspension were mixed.

Lemon leaves were gently pricked with a sterile needle (two wounds per leaf) to facilitate inoculation. A 100 μl aliquot of the treatment or control mixture was injected into each wound using a 1 ml sterile syringe. The inoculated seedlings were placed in a growth chamber with controlled conditions (24 °C, 75% relative humidity, 16 h light/8 h dark cycle, approximately 180 μmol photon m^−^^2^ s^−^^1^ light intensity) and incubated for 15 days. The incidence of citrus canker symptoms (yellow halos, callus-like eruptions) on the leaves was recorded daily, and the severity of the disease was evaluated based on the diameter of lesions.

#### 2.3.2 Biocontrol efficacy of JDF1 cell-free supernatant

Strain JDF1 was cultured in LB liquid medium at 28 °C with shaking (180 rpm) until OD_600_ reached 1.0. The bacterial culture was centrifuged at 12000 rpm for 15 min at 4 °C, and the supernatant was collected. The supernatant was filtered through a 0.22 μm sterile filter membrane (Millipore, USA) to obtain the cell-free supernatant. For the treatment group, equal volumes of JDF1 cell-free supernatant and *Xcc* suspension (1.0 × 10^8^ CFU/ml) were mixed; the control group was prepared as described in Section 2.3.1. The inoculation and incubation procedures were identical to those in Section 2.3.1, and the biocontrol efficacy was evaluated based on symptom development and GFP fluorescence intensity.

### 2.4 Thermostability assay of JDF1

Strain JDF1 was cultured in LB liquid medium until OD_600_ reached 1.0. The bacterial suspension was divided into aliquots and treated in a 50 °C water bath for 4 h or 6 h. After heat treatment, the suspension was cooled to room temperature. The biocontrol efficacy of the heat-treated JDF1 suspension against *Xcc* was evaluated using the same method as described in Section 2.3.1.

To further quantify the inhibitory effect of heat-treated JDF1 on *Xcc*, the procedures for plant sample selection and experimental treatment are detailed in Section 2.3.1. The leaf disk were surface-sterilized with 75% ethanol for 3 min, rinsed 3–4 times with sterile distilled water, and homogenized in 1 mL of sterile distilled water. Serial dilutions of the homogenate were spread onto LB agar plates supplemented with 50 μg/ml kanamycin (to select for *Xcc*, which was previously marked with a kanamycin resistance gene). The plates were incubated at 28 °C for 36–48 h, and the number of *Xcc* colonies was counted to determine the *Xcc* concentration in the leaves.

### 2.5 Genome sequencing and analysis of JDF1

#### 2.5.1 Genomic DNA extraction and quality control

Genomic DNA of JDF1 was extracted using the QIAGEN Genomic-tip 100/G Kit (QIAGEN, Germany) according to the manufacturer's instructions. The concentration of the extracted DNA was measured using a TBS 380 fluorometer (Turner BioSystems, USA), and the purity was determined using a NanoDrop 2000 spectrophotometer (Thermo Fisher Scientific, USA). Only DNA samples without degradation (verified by agarose gel electrophoresis), an OD_260_/OD_280_ ratio of 1.8–2.0, and a total amount of at least 15 μg were used for subsequent sequencing.

#### 2.5.2 Library construction and single-molecule real-time (SMRT) sequencing

Genomic DNA was fragmented into 8–10 kb segments using G-tubes (Covaris, USA). The fragmented DNA was end-repaired to generate blunt ends, and hairpin adapters were ligated to both ends to form a “SMRT Bell” library (Pacific Biosciences, Menlo Park, CA, USA). The library was annealed to a sequencing primer and bound to DNA polymerase, then loaded onto a Zero-Mode Waveguide (ZMW) chip. Sequencing was performed using the PacBio Sequel II platform (Pacific Biosciences, Menlo Park, CA, USA) with the Sequel II Binding Kit 2.0 and Sequel II Sequencing Kit 2.0 (Pacific Biosciences, Menlo Park, CA, USA). For specific steps, refer to Wang et al. (2025).

#### 2.5.3 Genome assembly and annotation

Raw sequencing reads were filtered to remove low-quality reads (quality score < 20) and adapter sequences using SMRT Link v10.1 software (Pacific Biosciences, Menlo Park, CA, USA). The filtered reads were assembled into a circular genome using the Hierarchical Genome Assembly Process (HGAP) v4.0. Gene prediction was performed using Prodigal v2.6.3. Functional annotation of the predicted genes was conducted by comparing sequences against public databases, including the Clusters of Orthologous Groups of proteins (COG) database, the Kyoto Encyclopedia of Genes and Genomes (KEGG) database, and the Carbohydrate-Active enZymes (CAZy) database. Biosynthetic gene clusters (BGCs) for secondary metabolites were predicted using antiSMASH v6.1.1.

### 2.6 Metabolomic analysis

Metabolite extraction and profiling workflow: LC-MS analytical conditions, data processing, and metabolite identification for JDF1 cell samples (100 mg, *n* = 3) refer to Peng et al. (2025). Samples were processed in 2 ml tubes with a 6 mm grinding bead.

### 2.7 Statistical analysis

All experiments were performed in triplicate, and the data were expressed as the mean ± standard deviation (SD). Significant differences among treatments were determined using one-way analysis of variance (ANOVA) followed by Duncan's multiple range test (*p* < 0.05). Figures were generated using GraphPad Prism 9.0 software (GraphPad Software, USA).

## 3 Results

### 3.1 Isolation and antagonistic function identification of biocontrol bacteria JDF1

**Figure 1:**
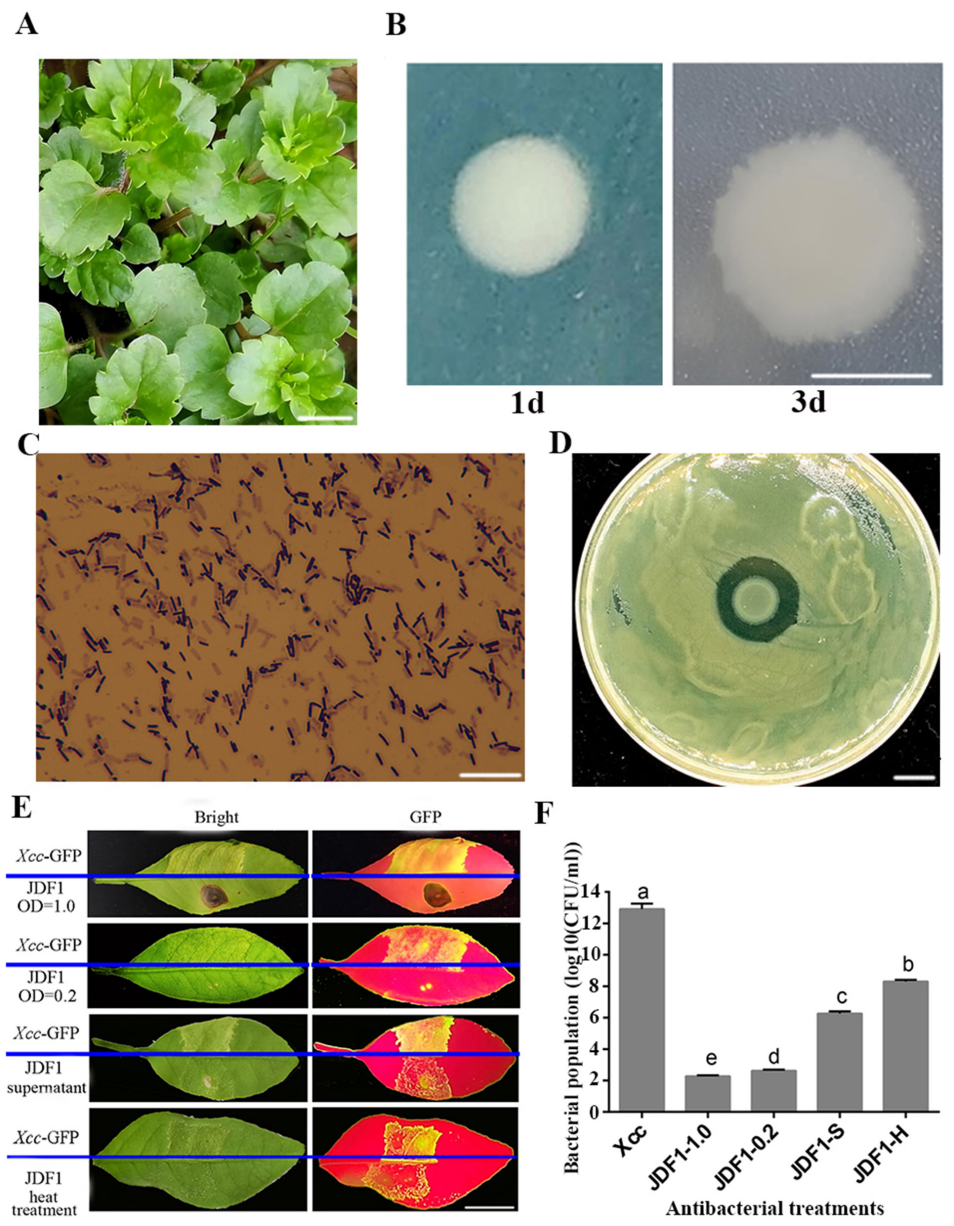
Isolation and antagonistic function identification of biocontrol bacteria JDF1. **(A)** *Veronica persica* plant. Bar = 1 cm. **(B)** Colonial morphology of JDF1 after 1-day and 3-day incubation. Bar = 1 cm. **(C)** Gram staining of JDF1. Bar = 200 μm. **(D)** JDF1 against *Xanthomonas citri* subsp. *citri* (*Xcc*) assessed by plate antagonism assay. **(E)** Control efficacy of JDF1 suspensions (OD_600_ = 1.0 and OD_600_ = 0.2), heat-treated JDF1 (50 °C, 6 h), and cell-free supernatant of JDF1 against citrus canker on leaves of “Eureka” lemon. Bar = 1 cm. **(F)** Bacterial titers of *Xcc* in lemon leaves following different treatments. Bar = 1 cm.

JDF1, an endophytic bacterium isolated from the leaves of *Veronica persica*, forms white and semi-translucent monocultural colonies with irregular and serrated edges (Figures 1A, B). Gram staining analysis (Figure 1C) revealed that the cells are rod-shaped with blunt ends, occur singly or in chains, and stain purple, indicating that they are Gram-positive. Using the plate confrontation assay, JDF1 was found to exhibit an antagonistic effect against *Xcc*, the pathogen responsible for citrus canker (Figure 1D). To evaluate the control efficacy of strain JDF1 against citrus canker, an inoculation experiment was conducted using seedlings of the susceptible variety Eureka lemon. The results showed that the control group exhibited typical symptoms of citrus canker, such as yellow halos and callus-like eruptions. In contrast, leaves injected with the JDF1 bacterial suspension showed no obvious canker symptoms. However, a high concentration (OD_600_ = 1.0) of the JDF1 suspension induced necrosis around the wound sites, while a low concentration (OD_600_ = 0.2) did not cause such necrosis, indicating that the JDF1 suspension provides effective disease control at low concentrations without causing phytotoxicity. After prolonged treatment at 50 °C, the antibacterial activity of the JDF1 fermentation broth decreased but remained significant (Figure 1E). Furthermore, the supernatant of the JDF1 culture was shown to alleviate citrus canker symptoms, suggesting that it may secrete antibacterial compounds that inhibit the proliferation of *Xcc*. Based on green fluorescence observation and *Xcc* plate counting, the ranking of control efficacy was as follows: JDF1 suspension at OD_600_ = 1.0 > JDF1 suspension at OD_600_ = 0.2 > JDF1 supernatant > JDF1 suspension treated at 50 °C for 6 h (Figures 1E, F).

### 3.2 Genomic sequencing and homologous alignment analysis of JDF1

**Figure 2:**
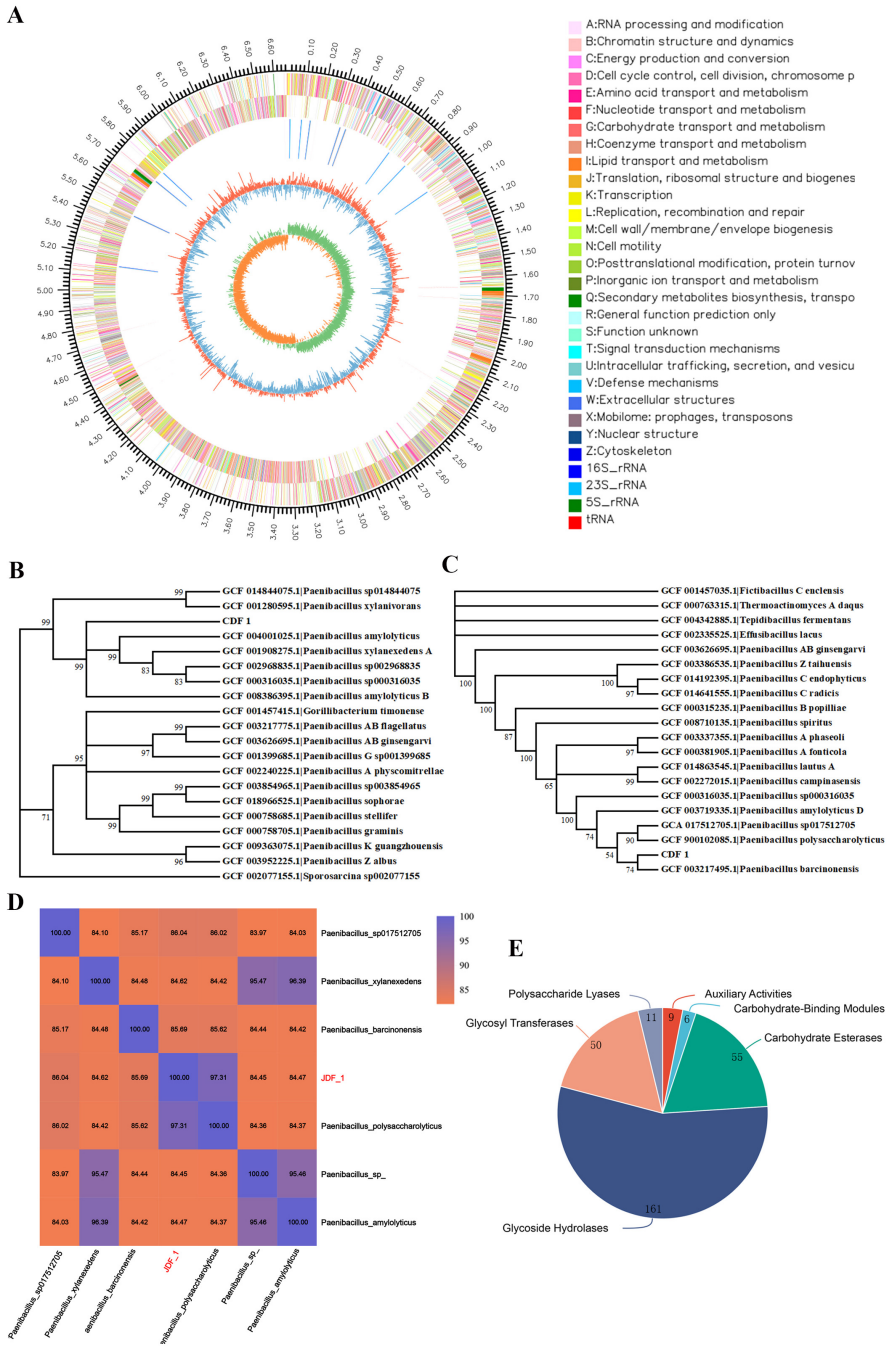
Genomic analysis of JDF1. **(A)** Circos plot of the JDF1 genome. From outer to inner, the circles represent: genome scale markers, genes on the forward strand, genes on the reverse strand, ncRNAs, GC content, and GC skew. **(B)** Phylogenetic analysis based on 16S rRNA gene sequences. The 19 most closely related strains at the genus and species levels were selected, and a phylogenetic tree was constructed using the neighbor-joining (NJ) method in MEGA 6.0. **(C)** Phylogenetic analysis based on housekeeping genes. A total of 31 housekeeping genes (*dnaG, frr, infC, nusA, pgk, pyrG, rplA, rplB, rplC, rplD, rplE, rplF, rplK, rplL, rplM, rplN, rplP, rplS, rplT, rpmA, rpoB, rpsB, rpsC, rpsE, rpsI, rpsJ, rpsK, rpsM, rpsS, smpB, tsf* ) from 20 strains of the same genus were used to construct the phylogenetic tree via the neighbor-joining (NJ) method in MEGA. **(D)** Average Nucleotide Identity (ANI) analysis of JDF1. **(E)** Statistical chart of carbohydrate-active enzyme annotations. The pie chart illustrates the relative proportions of different enzyme categories, with colors representing distinct families and numbers indicating the count of associated proteins.

Sequencing analysis revealed that the JDF1 genome comprises a single chromosome without plasmids. A circular genomic map was constructed showing that the genome has a total length of 6,670,835 bp with a GC content of 45.78 mol% and 5,788 genes (Figure 2A and Supplementary Table S1). The 16S rRNA gene sequence of the biocontrol strain JDF1 was obtained through sequencing, with a length of 1,480 bp. BLAST analysis against the GenBank database indicated that the 16S rRNA gene sequence of JDF1 shares the highest homology with sequences from *Paenibacillus* spp. A phylogenetic tree constructed using 16S rRNA gene sequences from 19 representative *Paenibacillus* strains showed that JDF1 shares the highest similarity with *Paenibacillus xylanexedens* A (98.52%) and *Paenibacillus amylolyticus* (98.46%; Figure 2B and Supplementary Table S2). Additionally, a phylogenetic tree based on 31 housekeeping genes from the JDF1 genome demonstrated that JDF1 is most closely related to *Paenibacillus polysaccharolyticus* (99.8%) and *Paenibacillus barcinonensis* (98.9%; Figure 2C and Supplementary Table S3). However, despite these analyses, the species-level identification of JDF1 could not be definitively determined based on either 16S rRNA or housekeeping gene phylogenies. Furthermore, ANI analysis results indicated that JDF1 belongs to the same species as *P. polysaccharolyticus*, with a similarity of 97.31% (Figure 2D). Meanwhile, based on the above homology analyses, we speculate that JDF1 may possess carbohydrate metabolic functions. Prediction of its protein-encoding genes revealed that the strain likely contains a variety of carbohydrate-active enzymes (CAZymes), including glycoside hydrolases (GHs; 161), glycosyltransferases (GTs; 50), polysaccharide lyases (PLs; 11), carbohydrate esterases (CEs; 55), carbohydrate-binding modules (CBMs; 6), and auxiliary activities (AAs; 9; Figure 2E and Supplementary Table S4). These findings further support the results of the homology analysis of JDF1.

### 3.3 Gene function and metabolic pathway analysis of JDF1

**Figure 3:**
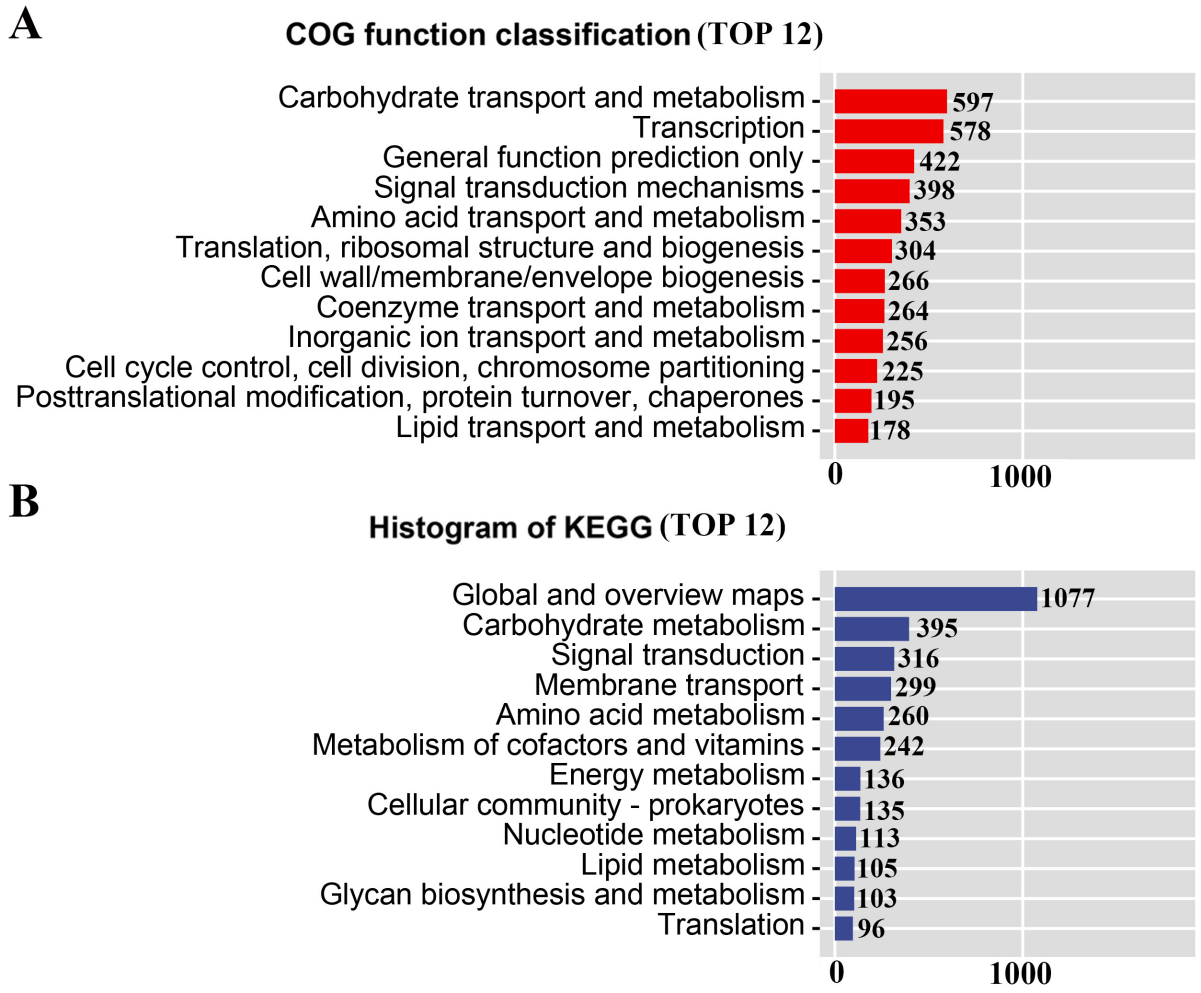
Gene function analysis of JDF1. **(A)** COG annotation of JDF1. **(B)** KEGG annotation of JDF1.

Comparison of the protein sequences of JDF1 against the Clusters of Orthologous Groups of proteins (COG) database showed that a total of 4,452 proteins were annotated. The major functional categories included carbohydrate transport and metabolism (597), transcription (578), signal transduction mechanisms (398), amino acid transport and metabolism (353), translation, ribosomal structure and biogenesis (304), and defense mechanisms (150; Figure 3A and Supplementary Table S5). KEGG annotation was performed to systematically analyze the functional roles of genes in metabolic pathways in the JDF1 genome. The results showed that a total of 3,986 genes were annotated to KEGG pathways, including carbohydrate metabolism (395), signal transduction (316), membrane transport (299), amino acid metabolism (260), and metabolism of cofactors and vitamins (242; Figure 3B and Supplementary Table S6).

### 3.4 Virulence factors and drug resistance analysis of JDF1

**Figure 4:**
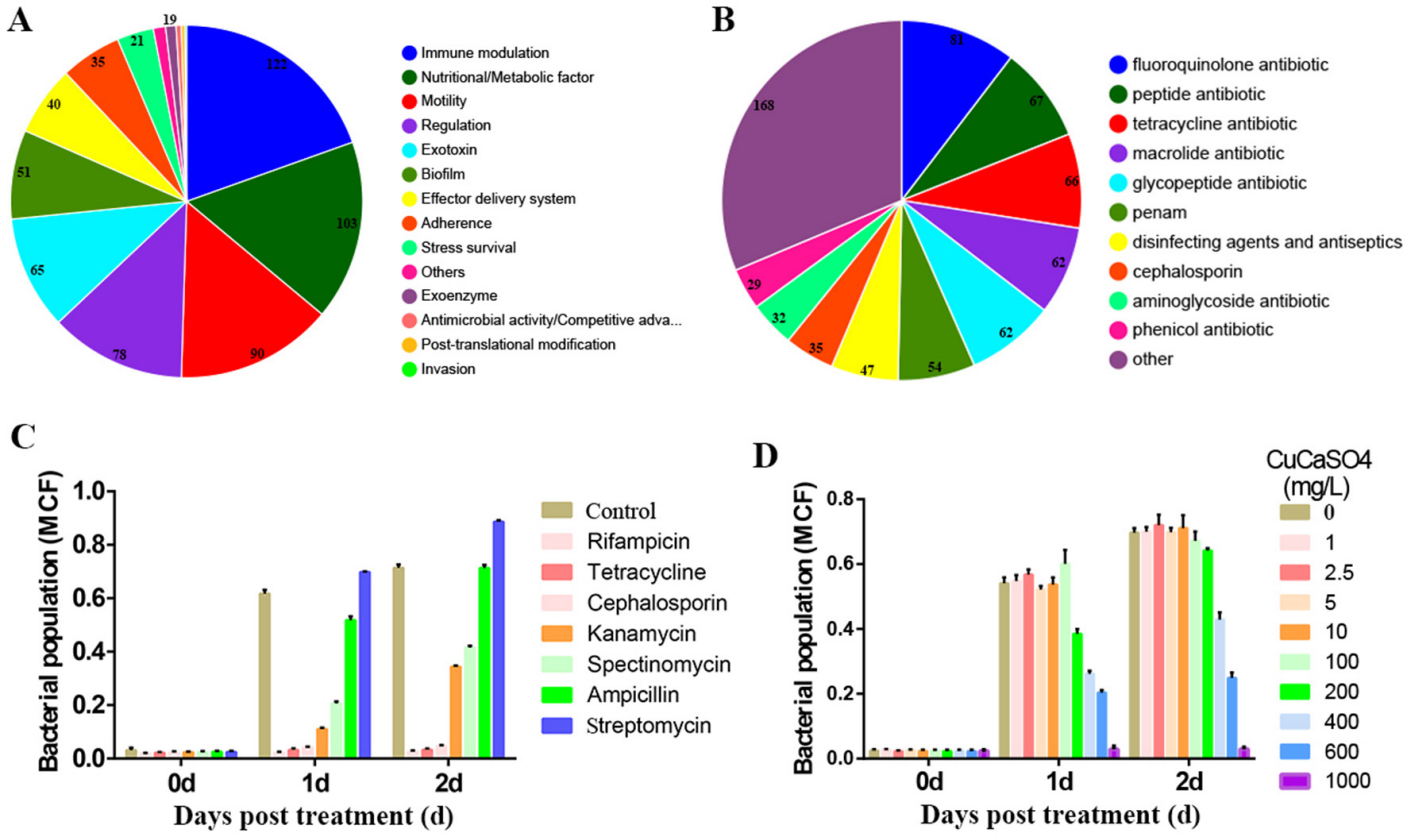
Virulence factors and drug resistance analysis of JDF1. **(A)** Prediction and statistics of virulence genes. The text above each pie/doughnut chart indicates the primary category of virulence factors, while the text to the right of each chart specifies the secondary category. Different colors in the charts represent different secondary categories, with the area of each segment indicating the relative proportion of genes in that category. Numbers represent gene counts. **(B)** Prediction and statistics of antibiotic resistance genes. Different colors in the pie/doughnut chart represent various drug classes, with the area of each segment indicating the relative proportion of genes in that category. Numbers represent gene counts. **(C)** Evaluation of antibiotic resistance in JDF1. **(D)** Evaluation of copper tolerance in JDF1. MCF, McFarland Unit.

Previous studies indicated that a high concentration of JDF1 bacterial suspension can cause necrosis in citrus leaves. Therefore, we predicted the virulence factors encoded in the JDF1 genome. The results revealed the presence of various virulence factor synthesis genes, primarily including immune modulation (122), nutritional/metabolic factor (103), motility (90), regulation (78), exotoxin (65), biofilm (51), and effector delivery system (40; Figure 4A and Supplementary Table S7). Meanwhile, prediction of antibiotic resistance genes in JDF1 identified 37 potential genes conferring resistance to various antimicrobial agents, including fluoroquinolone, peptide antibiotic, tetracycline, macrolide, glycopeptide, penam, disinfecting agents and antiseptics, cephalosporin, aminoglycoside, phenicol, rifamycin, carbapenem, cephamycin, oxazolidinone, monobactam, lincosamide, aminocoumarin, streptogramin and glycylcycline (Figure 4B and Supplementary Table S8). To evaluate the antibiotic resistance of strain JDF1, seven commonly used antibiotics were selected for testing. The results indicated that JDF1 was resistant to ampicillin and streptomycin, and showed intermediate resistance to kanamycin and spectinomycin. In contrast, the growth of JDF1 was completely inhibited in the presence of rifampicin, tetracycline, and cephalosporin (Figure 4C). Copper-based agents are the most common chemical bactericides for controlling citrus canker, with a typical field application concentration of approximately 1–2 g/L. To evaluate the potential of combining the biocontrol agent JDF1 with copper formulations in field applications, we assessed its copper tolerance. The results showed that the growth of JDF1 was not affected at concentrations of CaCuSO_4_ below 200 mg/L. At 400 mg/L, JDF1 growth was reduced to 60% of the control, and further declined to 40% at 600 mg/L. Complete inhibition of growth was observed at 1,000 mg/L (Figure 4D). These findings indicate that JDF1 exhibits a high level of copper tolerance, suggesting its potential compatibility with copper-based agents in integrated control strategies.

### 3.5 Antibacterial substances analysis of JDF1

**Figure 5:**
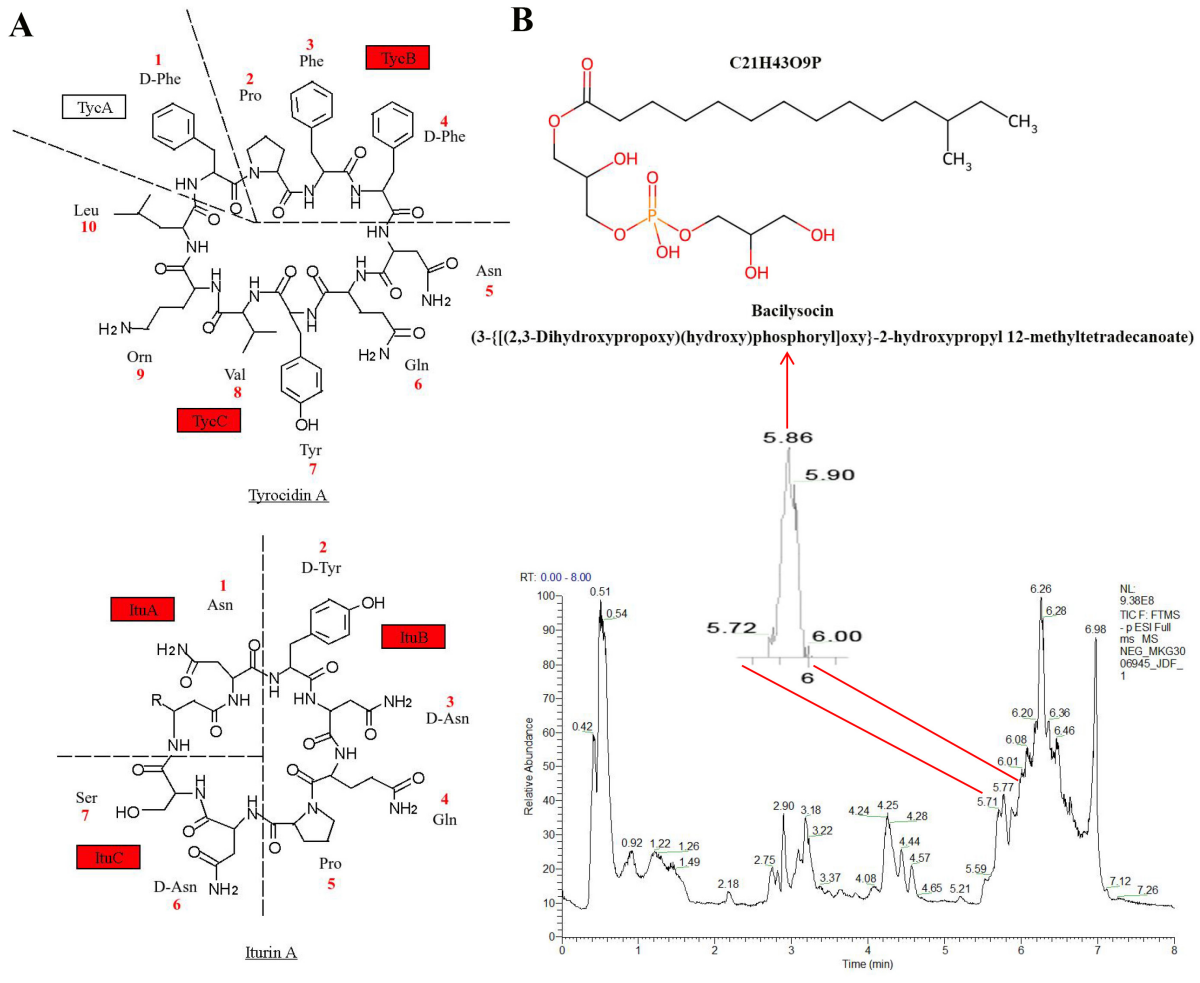
Analysis of antimicrobial metabolites from JDF1. **(A)** Two potential polypeptide antimicrobial substances were identified based on genomic analysis of JDF1. The red box indicates that the gene has been annotated in the genome. **(B)** The antimicrobial metabolite bacilysocin was detected in the metabolome of JDF1.

Genomic annotation identified JDF1 genes associated with antibiotic biosynthesis, categorized into ribosomally synthesized peptides (RPS) and non-ribosomally synthesized peptides (NRPS). For non-ribosomal antibiotics, five genes were related to polyketide and terpenoid biosynthesis, and nine genes were associated with other NRPS pathways. The ribosomally synthesized antibiotics included two genes encoding class II lanthipeptide, five genes for ranthipeptide, one for proteusin and three for lassopeptide. Additionally, three siderophore biosynthesis genes were identified in the JDF1 genome (Supplementary Table S9). Further analysis revealed that nine NRPS pathway genes are involved in the synthesis of two antimicrobial peptides, including tyrocidin A and iturin A (Figure 5A). Additionally, a potential antimicrobial substance, bacilysocin, was detected in the metabolome (Figure 5B and Supplementary Table S10).

## 4 Discussion

Citrus bacterial canker poses a persistent threat to global citrus production, with existing control measures plagued by limitations such as pathogen resistance, environmental pollution, and long breeding cycles (Shahbaz et al., 2023). In this study, we isolated and characterized a novel thermotolerant endophytic *Paenibacillus strain* JDF1, which exhibits robust and multifaceted biocontrol potential against CBC. Notably, JDF1 retains over 60% of its antimicrobial activity after 6 h of heat treatment at 50 °C, addressing a critical limitation of the thermal instability of microbial agents that leads to their inefficacy in the field.

In greenhouse assays, low-concentration JDF1 (OD_600_ = 0.2) completely eliminated CBC symptoms on “Eureka” lemon without phytotoxicity, while high-concentration JDF1 (OD_600_ = 1.0) induced mild necrosis. The antibacterial mechanism of JDF1 is significantly different from that of its closely related genus, *Bacillus*, as *Bacillus* treatment does not induce a hypersensitivity response (Rabbee and Baek, 2023). These results indicate that JDF1 itself or its secretions can induce systemic acquired resistance (SAR) in citrus, which may involve salicylic acid-related signaling pathways (Zhang et al., 2010; Xiao et al., 2025). This also suggests that JDF1 has the potential to be developed as a SAR inducer, which represents an alternative approach for controlling citrus canker (Graham and Myers, 2013, 2016). Additionally, JDF1's cell-free supernatant suppresses *Xcc* proliferation, indicating that secondary metabolites contribute to its biocontrol activity—a trait shared with other effective BCAs such as *Bacillus amyloliquefaciens* F9, whose cell-free supernatant inhibits *Xcc* via lipopeptide extract components (Wang et al., 2022).

JDF1 is phylogenetically close to *P. xylanexedens* (98.52% 16S rRNA gene similarity) and *P. polysaccharolyticus* (99.8% housekeeping gene similarity and 97.31% ANI). Based on the above, we speculate that it is more likely to be a strain of *P. polysaccharolyticus*. Functional annotation based on the COG database and pathway analysis based on KEGG identified carbohydrate transport and metabolism (597 genes) and carbohydrate metabolism (395 genes) as the primary categories, respectively. These results demonstrate that JDF1 possesses polysaccharide-degrading capabilities, functionally similar to the previously reported *P. polysaccharolyticus* sp., exhibiting multiple hydrolytic enzyme activities such as xylanolytic and cellulolytic activities (Madhaiyan et al., 2017; Hwang et al., 2021).

Notably, a relatively high concentration of JDF1 bacterial suspension induces necrosis in citrus leaves, which is attributed to the various virulence factor synthesis genes predicted in its genome (Figure 4A and Supplementary Table S7). Furthermore, we detected a variety of polyamine metabolites in the metabolome of JDF1, including cadaverine, spermine, and spermidine. Polyamines are considered a significant source of hydrogen peroxide (H_2_O_2_), which can trigger the hypersensitive response and the activation of defense-related genes. Consequently, polyamines play a crucial role in establishing plant resistance to specific diseases. Overexpression of polyamine biosynthetic genes can regulate endogenous polyamine levels. Based on this, the apple spermidine synthase gene *MdSPDS1* was introduced into sweet orange (*Citrus sinensis* Osbeck “Anliucheng”), and significantly reduced the susceptibility of the transgenic plants to citrus canker (Fu et al., 2011; Yoda et al., 2009). In summary, the necrotic lesions induced by high concentrations of JDF1 are likely the result of multiple factors, including the toxic effects caused by various virulence factors and the hypersensitive response triggered by polyamines. However, the induction of necrotic lesions by JDF1 appears to require extremely high concentrations and occurs only in a very limited area around wounds. These stringent triggering conditions make its occurrence almost negligible in practical applications. Therefore, the necrotic response induced by high-concentration JDF1 can be excluded from its mechanism of action against citrus canker. Its low concentration is already effective in preventing and controlling citrus canker, making it an effective and safe approach.

The JDF1 genome harbors 405 potential antibiotic resistance genes spanning 11 antimicrobial classes and exhibits phenotypic resistance to ampicillin and streptomycin, intermediate resistance to kanamycin and spectinomycin, and sensitivity to rifampicin, tetracycline, and cephalosporin. This resistance profile is strategically valuable for integrated disease management: streptomycin resistance allows JDF1 to coexist with streptomycin applications (a common emergency control for CBC outbreaks), while sensitivity to rifampicin provides a safety mechanism for targeted elimination if needed. Most notably, JDF1 tolerates CaCuSO_4_ at concentrations up to 600 mg/L. Given that the typical field application concentration of copper-based fungicides ranges from 1–2 g/L, this finding suggests potential for the combined field application of JDF1 and copper-based fungicides (Behlau et al., 2012).

The JDF1 genome contains 28 BGCs for antimicrobial metabolites, including polyketide, terpenoid, siderophore, tyrocidin A, iturin A, lanthipeptide (class II), ranthipeptide, and lassopeptide. Polyketides and terpenoids represent two major classes of important antibiotic sources, including macrolides, tetracyclines, monoterpenes, and diterpenes, which exhibit inhibitory activity against a wide range of pathogenic bacteria (Vasudevan and Lee, 2020; Zhang et al., 2024). However, no polyketide or terpenoid antibiotics were detected in the metabolome of JDF1 (with a Fragmentation Score ≥80). Siderophores are low-molecular-weight compounds secreted by bacteria or fungi that exhibit extremely high affinity for ferric iron (Fe^3+^). Their primary function is to scavenge scarce free iron from the environment, thereby depriving competitors (such as pathogenic bacteria) of this essential element and inhibiting their growth (Pradhan et al., 2023; Zang et al., 2025). Lanthipeptide antibiotics are key antimicrobial secondary metabolites produced by many biocontrol strains (Xu et al., 2020). Their core antibacterial mechanism involves high-affinity binding to lipid II, an essential precursor of bacterial cell wall synthesis, thereby achieving a dual bactericidal effect through “inhibition of cell wall biosynthesis” and “formation of transmembrane pores”. This unique multi-target mode of action makes them a highly important and promising class of antimicrobial agents (Moreira et al., 2024). Radical non-α-carbon thioether peptides (ranthipeptides) are a newly described class of ribosomally synthesized and post-translationally modified peptides (RiPPs). However, their antibacterial function and mechanism remain unclear (Precord et al., 2019). Lasso peptides are a class of ribosomally synthesized and post-translationally modified peptides (RiPPs) defined by their structurally rigid knotted fold, which confers bioactivity. Specifically, lariocidin (LAR) and lariocidin B (LAR-B) from *Paenibacillus* spp. M2 are such peptides that possess broad-spectrum activity against various bacterial pathogens. Their mechanism of action involves ribosomal binding, leading to the inhibition of bacterial growth by disrupting protein synthesis (Jangra et al., 2025). Tyrocidine A, a cyclic decapeptide, functions by disrupting cell membrane integrity and demonstrates broad-spectrum efficacy against Gram-positive bacteria as well as antifungal activity (Yang and Yousef, 2018). Iturin A is a class of highly important lipopeptide antibiotics, primarily produced by strains of the *Bacillus* genus, renowned for their potent antifungal activity. It functions by interacting with sterols (such as ergosterol in fungal membranes) to form transmembrane pores, leading to increased membrane permeability and cell death. It typically exhibits weak or no activity against bacteria, including Gram-positive bacteria, due to the absence of sterols—a key target—in bacterial cell membranes. Therefore, our results indicate that strain JDF1 has the potential to control fungal diseases, which is consistent with the established finding that iturin A exhibits potent antifungal activity (Zhang et al., 2025). Furthermore, LC-MS analysis identified a phospholipid antibiotic—bacilysocin—in the JDF1 strain, which exhibits significant antimicrobial activity against *S. aureus, Saccharomyces cerevisiae, Candida pseudotropicalis*, and *Cryptococcus neoformans*, but demonstrates no efficacy against most Gram-positive bacteria. However, its inhibitory effect on citrus canker has not been reported (Tamehiro et al., 2002). These results preliminarily reveal the potential antimicrobial substances produced by JDF1, thereby laying a theoretical foundation for its application in agricultural disease control and production cultivation.

Despite JDF1's promising biocontrol potential, several limitations must be addressed to accelerate its translational application: first, the relative contribution of individual secondary metabolites to JDF1's antagonistic activity remains unquantified. Second, JDF1's storage stability and field efficacy require further evaluation, despite its thermotolerance (retaining >60% activity at 50 °C). Third, JDF1's impact on the citrus phyllosphere microbiome—a key determinant of long-term disease suppression—has not been investigated. Finally, JDF1's compatibility with other CBC control measures requires long-term testing. While its copper/antibiotics tolerance enables integration with copper/antibiotics formulations, the measures need further evaluation. Overall, in our future research, the combined control of citrus canker in the field using JDF1 with agents such as copper-based formulations, as well as the isolation and identification of antibacterial substances, will be the focus of our investigations.

## 5 Conclusion

*Paenibacillus polysaccharolyticus* strain JDF1 represents a promising biocontrol agent (BCA) for controlling citrus bacterial canker (CBC), with unique advantages including thermostability, low phytotoxicity, dual mechanisms of action (direct antibiosis and induced systemic resistance, ISR), and compatibility with copper-based agents. Its genomic and metabolomic profiles reveal a rich repertoire of antimicrobial metabolites and adaptive traits, supporting its efficacy *in vitro* and in greenhouse assays. Addressing the identified limitations through targeted gene editing, formulation optimization, field trials, and microbiome analysis will further enhance JDF1's commercial potential, providing an eco-friendly alternative to chemical controls and advancing sustainable citrus production.

## Data Availability

The datasets presented in this study can be found in online repositories. The names of the repository/repositories and accession number(s) can be found in the article/Supplementary material.

## References

[B1] Ahmad A. A. Askora A. Kawasaki T Fujie M. Yamada T. (2014). The filamentous phage XacF1 causes loss of virulence in *Xanthomonas axonopodis* pv. citri, the causative agent of citrus canker disease. Front. Microbiol. 5:321. doi: 10.3389/fmicb.2014.00321 25071734 PMC4076744

[B2] Behlau F. Jones J. B. Myers M. E. Graham J. H. (2012). Monitoring for resistant populations of *Xanthomonas citri* subsp. *citri* and epiphytic bacteria on citrus trees treated with copper or streptomycin using a new semi-selective medium. Eur. J. Plant Pathol. 132, 259–270. doi: 10.1007/s10658-011-9870-7

[B3] Carvalho C. R. Dias A. C. Homma S. K. Cardoso E. J. (2020). Phyllosphere bacterial assembly in citrus crop under conventional and ecological management. Peer J. 8:e9152. doi: 10.7717/peerj.9152 32547860 PMC7274167

[B4] Costa A. Corallo B. Amarelle V. Stewart S. Pan D. Tiscornia S. . (2022). *Paenibacillus sp*. Strain UY79, isolated from a root nodule of *Arachis villosa*, displays a broad spectrum of antifungal activity. Appl. Environ. Microbiol. 88, e01645–e01621. doi: 10.1128/AEM.01645-21 34757818 PMC8788682

[B5] Fu X. Z. Chen C. W. Wang Y. Liu J. H. Moriguchi T. (2011). Ectopic expression of MdSPDS1 in sweet orange (*Citrus sinensis* Osbeck) reduces canker susceptibility: involvement of H_2_O_2_ production and transcriptional alteration. BMC Plant Biol. 11:55. doi: 10.1186/1471-2229-11-55 21439092 PMC3078878

[B6] Graham J. H. Gottwald T. R. Cubero J. Achor D. S. (2004). *Xanthomonas axonopodis* pv. citri: factors affecting successful eradication of citrus canker. Mol. Plant Pathol. 5, 1–15. doi: 10.1046/j.1364-3703.2004.00197.x 20565577

[B7] Graham J. H. Myers M. E. (2013). Integration of soil applied neonicotinoid insecticides and acibenzolar-S-methyl for systemic acquired resistance (SAR) control of citrus canker on young citrus trees. Crop Prot. 54, 239–243. doi: 10.1016/j.cropro.2013.09.002

[B8] Graham J. H. Myers M. E. (2016). Evaluation of soil applied systemic acquired resistance inducers integrated with copper bactericide sprays for control of citrus canker on bearing grapefruit trees. Crop Prot. 90, 157–162. doi: 10.1016/j.cropro.2016.09.002

[B9] Hwang J. Shin S. C. Han J. W. Hong S. P. Min W. K. Chung D. . (2021). Complete genome sequence of *Paenibacillus xylanexedens* PAMC 22703, a xylan-degrading bacterium. Mar. Genomics. 55:100788. doi: 10.1016/j.margen.2020.100788 32563695

[B10] Jangra M. Travin D. Y. Aleksandrova E. V. Kaur M. Darwish L. Koteva K. . (2025). A broad-spectrum lasso peptide antibiotic targeting the bacterial ribosome. Nature 640, 1022–1030. doi: 10.1038/s41586-025-08723-7 40140562 PMC12497486

[B11] Jia H. Orbovic V. Jones J. B. Wang N. (2016). Modification of the PthA4 effector binding elements in type I *CsLOB1* promoter using Cas9/sgRNA to produce transgenic duncan grapefruit alleviating XccΔpthA4: dCsLOB1.3 infection. Plant Biotechnol. J. 14, 1291–1301. doi: 10.1111/pbi.12495 27071672 PMC11389130

[B12] Kenfaoui J. Dutilloy E. Benchlih S. Lahlali R. Ait-Barka E. Esmaeel Q. (2024). *Bacillus velezensis*: a versatile ally in the battle against phytopathogens-insights and prospects. Appl. Microbiol. Biot. 108:439. doi: 10.1007/s00253-024-13255-7 39145847 PMC11327198

[B13] Lebedeva J. Jukneviciute G. Cepaite R. Vickackaite V. Pranckutè R. Kuisiene N. (2021). Genome mining and characterization of biosynthetic gene clusters in two cave strains of *Paenibacillus* sp. Front. Microbiol. 11:612483. doi: 10.3389/fmicb.2020.612483 33505378 PMC7829367

[B14] Luo R. F. Zhang S. Y. Wu Y. X. Jiao Z. Y. Bao M. L. Lan Y. T. . (2026). *Bacillus velezensis* RF2 rescued from ritrus phyllosphere: dual mechanisms and broad-spectrum activity for controlling citrus bacterial canker. Microorganisms. 14:121. doi: 10.3390/microorganisms14010121 41597640 PMC12843974

[B15] Madhaiyan M. Poonguzhali S. Saravanan V. S. Duraipandiyan V. Al-Dhabi N. A. Kwon S. W. . (2017). *Paenibacillus polysaccharolyticus* sp. nov., a xylanolytic and cellulolytic bacteria isolated from leaves of Bamboo *Phyllostachys aureosulcata*. Int. J. Syst. Evol. Microbiol. 67, 2127–2133. doi: 10.1099/ijsem.0.001901 28671536

[B16] McManus P. S. Stockwell V. O. Sundin G. W. Jones A. L. (2002). Antibiotic use in plant agriculture. Annu. Rev. Phytopathol. 40, 443–465. doi: 10.1146/annurev.phyto.40.120301.093927 12147767

[B17] Michavila G. Adler C. de Gregorio P. R. Lami M. J. Caram Di Santo M. C. Zenoff A. M. . (2017). *Pseudomonas* protegens CS1 from the lemon phyllosphere as a candidate for citrus canker biocontrol agent. Plant Biol. 19, 608–617. doi: 10.1111/plb.12556 28194866

[B18] Moreira R. Yang Y. Luo Y. Gilmore M. S. van der Donk W. A. (2024). *Bibacillin* 1: a two-component lantibiotic from *Bacillus thuringiensis*. RSC Chem. Biol. 5, 1060–1073. doi: 10.1039/D4CB00192C 39268544 PMC11385697

[B19] Peng S. Qin Y. Li B. Pan G. Zhao W. Feng Y. . (2025). Selenium solubilization by *Bacillus* sp. S01: mechanistic insights and environmental implications in paddy soils. J. Hazard Mater. 498:139823. doi: 10.1016/j.jhazmat.2025.139823 40934586

[B20] Pradhan S. Choudhury A. Dey S. Hossain M. F. Saha A. Saha D. (2023). Siderophore-producing *Bacillus amyloliquefaciens* BM3 mitigate arsenic contamination and suppress *Fusarium* wilt in brinjal plants. J. Appl. Microbiol. 134:lxad217. doi: 10.1093/jambio/lxad217 37740438

[B21] Precord T. W. Mahanta N. Mitchell D. A. (2019). Reconstitution and substrate specificity of the thioether-forming radical s-adenosylmethionine enzyme in freyrasin biosynthesis. ACS Chem. Biol. 14, 1981–1989. doi: 10.1021/acschembio.9b00457 31449382 PMC7362900

[B22] Qian J. Zhang T. Tang S. Zhou L. Li K. Fu X. . (2021). Biocontrol of citrus canker with endophyte *Bacillus amyloliquefaciens* QC-Y. Plant Protect. Sci. 57, 1–13. doi: 10.17221/62/2020-PPS

[B23] Rabbee M. F. Baek K. H. (2023). Detection of antagonistic compounds synthesized by *Bacillus velezensis* against *Xanthomonas citri* subsp. *citri* by metabolome and RNA sequencing. Microorganisms. 11:1523. doi: 10.3390/microorganisms11061523 37375024 PMC10301053

[B24] Richard D. Boyer C. Vernière C. Canteros B. I. Lefeuvre P. Pruvost O. (2017). Complete genome sequences of six copper-resistant *Xanthomonas citri pv. citri* strains causing asiatic citrus canker, obtained using long-read technology. Genome Announc. 5:10. doi: 10.1128/genomeA.00010-17 28336584 PMC5364209

[B25] Rodrigues J. P. Peti A. P. F. Figueiró F. S. de Souza Rocha I. Junior V. R. A. Silva T. G. . (2018). Bioguided isolation, characterization and media optimization for production of lysolipins by actinomycete as antimicrobial compound against *Xanthomonas citri* subsp *citri*. Mol. Biol. Rep. 45, 2455–2467. doi: 10.1007/s11033-018-4411-5 30311124

[B26] Shahbaz E. Ali M. Shafiq M. Atiq M. Hussain M. Balal R. M. . (2023). Citrus canker pathogen, its mechanism of infection, eradication, and impacts. Plants. 12:123. doi: 10.3390/plants12010123 36616252 PMC9824702

[B27] Tamehiro N. Okamoto-Hosoya Y. Okamoto S. Ubukata M. Hamada M. Naganawa H. . (2002). Bacilysocin, a novel phospholipid antibiotic produced by *Bacillus subtilis* 168. Antimicrob. Agents Chemother. 46, 315–320. doi: 10.1128/AAC.46.2.315-320.2002 11796336 PMC127064

[B28] Tu Q. Chen J. Guo J. (2013). Control of postharvest blue mold of Nanfeng mandarin by application of strain YS-1 *Paenibacillus brasilensis*. J. Food Sci. 78, M868–E873. doi: 10.1111/1750-3841.12142 23668413

[B29] Vasudevan U. M. Lee E. Y. (2020). Flavonoids, terpenoids, and polyketide antibiotics: role of glycosylation and biocatalytic tactics in engineering glycosylation. Biotechnol. Adv. 41:107550. doi: 10.1016/j.biotechadv.2020.107550 32360984

[B30] Vater J. Niu B. Dietel K. Borriss R. (2015). Characterization of novel fusaricidins produced by *Paenibacillus* polymyxa-M1 using MALDI-TOF mass spectrometry. J. Am. Soc. Mass Spectrom. 26, 1548–1558. doi: 10.1007/s13361-015-1130-1 26100395

[B31] Vieira G. Purić J. Morão L. G. dos Santos J. A. Inforsato F. J. Sette L. D. . (2018). Terrestrial and marine Antarctic fungi extracts active against *Xanthomonas citri* subsp. *citri*. Lett. Appl. Microbiol. 67, 64–71. doi: 10.1111/lam.12890 29604211

[B32] Villamizar S. Ferro J. A. Caicedo J. C. Alves L. M. C. (2020). Bactericidal effect of entomo pathogenic bacterium *Pseudomonas entomophila* against *Xanthomonas citri* reduces citrus canker disease severity. Front. Microbiol. 11:1431. doi: 10.3389/fmicb.2020.01431 32670251 PMC7327231

[B33] Wang X. Liang L. Shao H. Ye X. Yang X. Chen X. . (2022). Isolation of the novel strain *Bacillus amyloliquefaciens* F9 and identification of lipopeptide extract components responsible for activity against *Xanthomonas citri* subsp. citri. Plants. 11:457. doi: 10.3390/plants11030457 35161438 PMC8840523

[B34] Wang Z. Du C. Yan R. Li S. Zheng G. Ding D. (2025). Sustainable polyhydroxybutyrate (PHB) production from biowastes by *Halomonas* sp. WZQ-1 under non-sterile conditions. Int. J. Biol. Macromol. 311:143643. doi: 10.1016/j.ijbiomac.2025.143643 40306522

[B35] Xiao Y. X. Xiao C. Tong Z. He X. J. Wang Z. Q. Zhang H. Y. . (2025). Four MES genes from calamondin (*Citrofortunella microcarpa*) regulated citrus bacterial canker resistance through the plant hormone pathway. Front. Plant Sci. 15:1513430. doi: 10.3389/fpls.2024.1513430 39902200 PMC11788333

[B36] Xu P. Xie S. Liu W. Jin P. Wei D. Yaseen D. G. . (2020). Comparative genomics analysis provides new strategies for bacteriostatic ability of *Bacillus velezensis* HAB-2. Front. Microbiol. 11:594079. doi: 10.3389/fmicb.2020.594079 33281792 PMC7705179

[B37] Yang X. Yousef A. E. (2018). Antimicrobial peptides produced by *Brevibacillus* spp.: structure, classification and bioactivity: a mini review. World J. Microbiol. Biotechnol. 34:57. doi: 10.1007/s11274-018-2437-4 29594558

[B38] Yi W. Chen C. Gan X. (2022). Polymyxin B_1_ and E_2_ from *Paenibacillus polymyxa* Y-1 for controlling rice bacterial disease. Front. Cell Infect. Microbiol. 12:866357. doi: 10.3389/fcimb.2022.866357 35419296 PMC8995708

[B39] Yoda H. Fujimura K. Takahashi H. Munemura I. Uchimiya H. Sano H. (2009). Polyamines as a common source of hydrogen peroxide in host-and nonhost hypersensitive response during pathogen infection. Plant Mol. Biol. 70, 103–112. doi: 10.1007/s11103-009-9459-0 19190986

[B40] Zang Z. Zhang C. Park K. J. Schwartz D. A. Podicheti R. Lennon J. T. . (2025). Streptomyces secretes a siderophore that sensitizes competitor bacteria to phage infection. Nat. Microbiol. 10, 362–373. doi: 10.1038/s41564-024-01910-8 39779880 PMC12107555

[B41] Zhang D. Huang K. Zou D. Wang B. Wu X. Ye C. . (2025). Enhanced iturin a synthesis and antifungal effect of *Bacillus amyloliquefaciens* by genetic engineering of protease, atp supply and itu operon. J. Agric. Food Chem. 73, 24092–24101. doi: 10.1021/acs.jafc.5c05592 40944921

[B42] Zhang J. Gao L. Lin H. Liang Y. You M. Ding L. . (2024). Discovery of antibacterial compounds against *Xanthomonas citri* subsp. *citri* from a marine fungus *Aspergillus terreus* SCSIO 41202 and the mode of action. J. Agric. Food Chem. 72, 12596–12606. doi: 10.1021/acs.jafc.4c02769 38771666

[B43] Zhang Y. Xu S. Ding P. Wang D. Cheng Y. T. He J. . (2010). Control of salicylic acid synthesis and systemic acquired resistance by two members of a plant-specific family of transcription factors. Proc. Natl. Acad. Sci. USA. 107, 18220–18225. doi: 10.1073/pnas.1005225107 20921422 PMC2964219

